# Hookworm Secreted Extracellular Vesicles Interact With Host Cells and Prevent Inducible Colitis in Mice

**DOI:** 10.3389/fimmu.2018.00850

**Published:** 2018-04-30

**Authors:** Ramon M. Eichenberger, Stephanie Ryan, Linda Jones, Geraldine Buitrago, Ramona Polster, Marcela Montes de Oca, Jennifer Zuvelek, Paul R. Giacomin, Lindsay A. Dent, Christian R. Engwerda, Matthew A. Field, Javier Sotillo, Alex Loukas

**Affiliations:** ^1^Centre for Biodiscovery and Molecular Development of Therapeutics, Australian Institute of Tropical Health and Medicine, James Cook University, Cairns, QLD, Australia; ^2^Immunology and Infection Laboratory, QIMR Berghofer Medical Research Institute, Brisbane, QLD, Australia; ^3^Pathology Queensland Cairns Laboratory, Queensland Health, Cairns, QLD, Australia; ^4^School of Biological Sciences, University of Adelaide, Adelaide, SA, Australia; ^5^Department of Immunology, John Curtin School of Medical Research, Australian National University, Canberra, ACT, Australia

**Keywords:** nematode, colitis, immunomodulation, parasite–host interaction, miRNA, proteomics, exosome, extracellular vesicles

## Abstract

Gastrointestinal (GI) parasites, hookworms in particular, have evolved to cause minimal harm to their hosts, allowing them to establish chronic infections. This is mediated by creating an immunoregulatory environment. Indeed, hookworms are such potent suppressors of inflammation that they have been used in clinical trials to treat inflammatory bowel diseases (IBD) and celiac disease. Since the recent description of helminths (worms) secreting extracellular vesicles (EVs), exosome-like EVs from different helminths have been characterized and their salient roles in parasite–host interactions have been highlighted. Here, we analyze EVs from the rodent parasite *Nippostrongylus brasiliensis*, which has been used as a model for human hookworm infection. *N. brasiliensis* EVs (*Nb*-EVs) are actively internalized by mouse gut organoids, indicating a role in driving parasitism. We used proteomics and RNA-Seq to profile the molecular composition of *Nb*-EVs. We identified 81 proteins, including proteins frequently present in exosomes (like tetraspanin, enolase, 14-3-3 protein, and heat shock proteins), and 27 sperm-coating protein-like extracellular proteins. RNA-Seq analysis revealed 52 miRNA species, many of which putatively map to mouse genes involved in regulation of inflammation. To determine whether GI nematode EVs had immunomodulatory properties, we assessed their potential to suppress GI inflammation in a mouse model of inducible chemical colitis. EVs from *N. brasiliensis* but not those from the whipworm *Trichuris muris* or control vesicles from grapes protected against colitic inflammation in the gut of mice that received a single intraperitoneal injection of EVs. Key cytokines associated with colitic pathology (IL-6, IL-1β, IFNγ, and IL-17a) were significantly suppressed in colon tissues from EV-treated mice. By contrast, high levels of the anti-inflammatory cytokine IL-10 were detected in *Nb*-EV-treated mice. Proteins and miRNAs contained within helminth EVs hold great potential application in development of drugs to treat helminth infections as well as chronic non-infectious diseases resulting from a dysregulated immune system, such as IBD.

## Introduction

Parasitic helminths (worms) modify the immune system of their host to avoid immune ejection, a strategy which promotes their long-term survival and results in chronic infection (1), but also has a bystander effect by protecting against the onset of inflammatory disorders that result from a dysregulated immune response (2). Hookworms, blood-feeding intestinal nematode parasites, are particularly adept at manipulating the immune systems of their mammalian hosts (3). Hookworm infection is one of the major human ailments affecting approximately 600 million people worldwide (4, 5). When hookworms first encounter a mammalian host, they release a suite of molecules referred to as excretory/secretory products (ESP), a mixture of proteins, carbohydrates, and lipids that represent the primary interface between hookworms and their hosts. In helminth parasites, the ES proteins orchestrate a wide range of activities crucial for their survival and propagation, including penetration of the host dermis, tissue invasion, feeding, reproduction, and evasion of the host immune system (3, 6, 7).

*Nippostrongylus brasiliensis* is a nematode of mice and rats, although it does infect a number of other rodent species (8). Because of its similarities to the life cycle of hookworm species (e.g., *Ancylostoma* spp., and *Necator americanus*), this species is often referred as the “rat hookworm” and has been frequently used as a model to study the immunobiology of human hookworm infections (9–13).

Following the migration of infective larvae (L3) through rodent tissues, *N. brasiliensis* triggers a highly polarized T helper type 2 (Th2) response in the skin, lungs, and intestinal mucosa (13), characteristics present also in human hookworm infections, including CD4+ T cell-dependent IgE production, eosinophilia, mastocytosis, and mucus production (3). Furthermore, hookworm infections are characterized by the generation of an immune-regulatory environment with the anti-inflammatory cytokines IL-10 and TGFβ, and regulatory T cells, type 2 innate lymphoid cells, tolerogenic dendritic cells, and M2 macrophages to prevent potentially dangerous pathology (14, 15).

Because of the exquisite immunomodulatory capacity of helminths, helminth therapy is under investigation for the treatment of inflammatory diseases, and has shown promise in both clinical trials and studies in animals with a range of inflammatory diseases, such as celiac disease, asthma, multiple sclerosis, and inflammatory bowel diseases (IBD) (16–20). Different research groups—including us—have demonstrated that the immunomodulatory environment induced by hookworms can be attributed to their secreted products (7, 21–25).

There is emerging evidence of the release of extracellular vesicles (EVs) during helminth infections—which correspond to a sub-fraction of the ESP—playing important roles in both parasite–parasite communications as well as in parasite–host interactions (26, 27). Nematode roundworm EVs can suppress potentially dangerous type 2 innate responses and eosinophilia and generate a regulatory and/or suppressive immune state that is beneficial for the parasite’s long-term survival (28). EVs have also been reported from platyhelminth flatworms (29, 30): schistosome EVs impact macrophage differentiation (31), and liver fluke EVs are internalized by human cholangiocytes and promote cell proliferation and potentially contribute to the development of liver cancer (32).

It was demonstrated that hookworm ESP mitigate colitis in different mouse models (21–23), and at least one recombinant ESP protein has been shown to possess anti-colitic properties (33). Here, we characterize the protein- and miRNA-cargo of secreted EVs from the hookworm-like nematode *N. brasiliensis*, show that these EVs are internalized by cells in murine gastrointestinal (GI) tract organoids, and evaluate their immunomodulatory properties in experimentally induced murine colitis. We then compared the data to that generated with EVs from a distantly related intestinal nematode, the whipworm *Trichuris muris* (*Tm*), and discuss the outcomes in terms of the immunobiology of these two major human helminth infections. This study conveys novel insights into the roles of nematode EVs and reveals potential applications of an entirely new generation of therapeutics to treat inflammatory disorders.

## Materials and Methods

### Parasite Material, Isolation of ESP, and EV Purification

Excretory/secretory products were collected from adult *N. brasiliensis* and *Tm* parasites, and EVs were purified. Exosome-like vesicles from grapes (“grapeosomes”) were purified and used as a negative purification and vesicle control.

*Nippostrongylus brasiliensis* was maintained in Sprague–Dawley rats (Animal Resources Centre, Perth, WA, Australia) as previously described (10). Infective L3 were prepared from 2-week rat fecal cultures. Adult worms were recovered from small intestines on day 8 post infection following subcutaneous injection of 3,000 infective L3. Adult worms were washed in PBS containing 5× antibiotic/antimycotic (AA; Sigma-Aldrich, St. Louis, MO, USA) and cultured in 24-well plates (500 worms/well) for 7 days in RPMI containing 1× AA and 1× GlutaMAX™ supplement (Gibco, Thermo Fisher, Waltham, MA, USA) at 37°C and 5% CO_2_. The media obtained during the first 4 h after parasite culturing was discarded. ESP were collected daily, subjected to sequential differential centrifugation at 500, 2,000, and 4,000 *g* for 30 min each to remove eggs and parasite debris. For the isolation of ES products, media was concentrated using a 10 kDa spin concentrator (Merck Millipore, Billerica, MA, USA) and stored at 1.0 mg/ml in PBS at −80°C until used.

*Trichuris muris* parasites were obtained from genetically susceptible B10.BR mice (Animal Resources Centre) infected with 200 *Tm* eggs. Adult worms were harvested from the cecum of infected mice 5 weeks after infection, washed in PBS containing 5× AA and cultured in 6-well plates for 5 days in RPMI containing 1× AA, at 37°C and 5% CO_2_. Each well contained ~500 worms in 4.5 ml media. Further processing was similar to that described herein for ESP from *N. brasiliensis*. Dead worms were removed and ES products were collected daily.

We chose exosomes derived from grapes as a control for our animal studies because they served as a non-mammalian source of EVs that are capable of being internalised by mouse organoid cells and protect against dextran sulfate sodium-induced colitis (34). Grapeosomes were purified from commercially purchased grapes (*Vitis vinifera* “Thompson seedless”) according to Ju et al. (34) with some modifications. Peeled grapes were minced and filtered through a 21 µm nylon mesh (Scrynel, Lanz-Anliker, Rohrbach, Switzerland) and 0.22 µm Steritop^®^ Membrane (GP Millipore Express^®^PLUS, Merck) and further processed as described herein for parasite ESP.

For the isolation of EVs, the media obtained after differential centrifugation was processed as described previously (30). Briefly, concentrated ESP were centrifuged for 45 min at 15,000 *g* to remove larger vesicles. A MLS-50 rotor (Beckman Coulter, Brea, CA, USA) was used to ultracentrifuge the supernatant for 3 h at 120,000 *g*. Supernatant resulting from this centrifugation corresponds to vesicle-depleted ESP (protein fraction). The resultant pellet was resuspended in 70 µl of PBS and subjected to Optiprep^®^ density gradient (ODG) separation. 1 ml of 40, 20, 10, and 5% iodixanol solutions prepared in 0.25 M sucrose, 10 mM Tris–HCl, pH 7.2, were layered in decreasing density in an ultracentrifuge tube, and the 70 µl containing the resuspended EVs was added to the top layer and ultracentrifuged at 120,000 *g* for 18 h at 4°C. 70 μl of PBS was added to the control tube prepared as described above. A total of 12 fractions were recovered from the ODG, and the excess Optiprep^®^ solution was removed by buffer exchanging with 8 ml of PBS containing 1× EDTA-free protease inhibitor cocktail (Santa Cruz, Dallas, TX, USA) using a 10 kDa spin concentrator. The absorbance (340 nm) was measured in each of the fractions and density was calculated using a standard curve with known standards. The protein concentration of all fractions was measured using a Pierce BCA Protein Assay Kit (Thermo Fisher). All fractions were kept at −80°C until use.

### Size and Concentration Analysis of EVs

The size distribution and particle concentration of fractions recovered after ODG were measured using tunable resistive pulse sensing (TRPS) by qNano (Izon, Christchurch, New Zealand) following the manufacturer’s instructions for working with smaller range nanopores. Voltage and pressure values were set to optimize the signal to ensure high sensitivity. A nanopore NP100 was used for all fractions analyzed except for the grape vesicles, where a NP150 was used. Calibration was performed using CP100 carboxylated polystyrene calibration particles (Izon) at a 1:1,000 dilution. Samples were diluted 1:5 and applied to the nanopore. The size and concentration of particles were determined using the software provided by Izon (version 3.2). Protein concentration was measured in all fractions, and EV purity determined as described previously (35).

### Proteomic Analysis

For the proteomic analysis of EVs from *N. brasiliensis*, 50 µg of protein of the ODG fractions with a density of 1.06–1.10 g/ml (fractions 7–9) were loaded on a 12% SDS-PAGE gel and electrophoresed at 100 V until the protein marker reached 2/3 of the total run length (approximately for 1.5 h). Each lane was sliced into 10 pieces, which were subjected to trypsin digestion as described previously (12). The final digest supernatant was removed from the gel slices, and residual peptides were removed from the gel slices by washing three times with 0.1% trifluoroacetic acid for 45 min at 37°C. Peptide samples were combined into 5 tubes per lane, resulting in total 15 samples for mass spectrometry analysis. Samples were desalted and concentrated using Zip-Tip^®^ and kept at −80°C until use.

Samples were reconstituted in 10 µl of 5% formic acid. Six microliters of sample was injected onto a 50 mm 300 µm C18 trap column (Agilent Technologies, Santa Clara, CA, USA) and desalted for 5 min at 30 µl/min using 0.1% formic acid (aq). Peptides were then eluted onto an analytical nano HPLC column (150 mm × 75 µm 300SBC18, 3.5 µm, Agilent Technologies) at a flow rate of 300 nl/min and separated using a 95 min gradient of 1–40% buffer B (90/10 acetonitrile/0.1% formic acid) followed by a steeper gradient of 40–80% buffer B in 5 min. The mass spectrometer (ABSCIEX 5600+) operated in information-dependent acquisition mode, in which a 1-s TOF MS scan from 350–1,400 *m*/*z* was performed, and for product ion ms/ms 80–1,400 *m/z* ions observed in the TOF-MS scan exceeding a threshold of 100 counts and a charge state of +2 to +5 were set to trigger the acquisition of product ion. Analyst 1.6.1 (ABSCIEX) software was used for data acquisition and analysis.

For the analysis of the EV mass spectrometry data, a database was built using the *N. brasiliensis* genome (PRJEB511) with the common repository of adventitious proteins (cRAP^1^) appended to it. Database search was performed using Mascot Versions 2.4 (Matrix Science Ltd., London, UK) and X!Tandem, MS-GF+, OMSSA, and Tide search engines using SearchGUI (36). The same parameters were used as described in Ref. (37).

The mass spectrometry proteomics data have been deposited in the ProteomeXchange Consortium *via* the PRIDE partner repository with the dataset identifiers PXD009165 and 10.6019/PXD009165. A final list of parasite-specific proteins resulted by combining the different fractions and removing hits for common contaminants from the cRAP database, considering only proteins containing at least two validated unique peptides matching *N. brasiliensis* gene models. Proteins were functionally classified according to Gene Ontology categories using the software Blast2GO basic version 4.0.7 (38). Putative signal peptides and transmembrane domain(s) were predicted using the programs CD-Search tool (39) and SignalP (40). Structural comparison of proteomic datasets was performed by all-vs-all blast in NCBI Blast + executables (v2.7.1).

### miRNA Analysis

Biological replicates of *N. brasiliensis* EVs (*Nb*-EVs) obtained from three different batches of worms were used. ODG fractions with a density between 1.07 and 1.09 (fractions containing pure EV samples after TRPS analysis) were pooled and excess Optiprep^®^ solution was removed by buffer exchanging. miRNA was extracted using the mirVana™ miRNA Isolation Kit (Thermo Fisher) according to the manufacturer’s instructions. RNA was eluted over two fractions of 50 µl each and stored at −80°C until analyzed.

The RNA quality, yield, and size of total and small RNAs were analyzed using capillary electrophoresis (Agilent 2100 Bioanalyzer, Agilent Technologies). miRNA was prepared for sequencing using a QIAseq™ miRNA library preparation kit(Qiagen, Hilden, Germany) according to the manufacturer’s instructions. RNA-Seq was performed on a NextSeq 500 (Illumina, single-end 75-bp SR mid output run, up to 130M reads per sample). Quality control, library preparation, and sequencing were performed at the Ramaciotti Centre for Genomics at the University of New South Wales. The data have been deposited in NCBI’s Gene Expression Omnibus under GEO series accession number GSE111478.

The miRDeep2 package (41) was used to identify known and putative novel miRNAs present in all miRNA replicates. As there are no *N. brasiliensis* miRNAs available in miRBase release 21 (42), the miRNAs from the nematodes *Ascaris suum, Brugia malayi, Caenorhabditis elegans, Caenorhabditis brenneri, Caenorhabditis briggsae, Caenorhabditis remanei, Haemonchus contortus, Pristionchus pacificus, Panagrellus redivivus*, and *Strongyloides ratti* were utilized as a training set for the algorithm. Only miRNA sequences commonly identified in all replicates were included for further analyses. The interaction between miRNA and murine host genes was predicted using the miRanda algorithm 3.3a (43). Input 3’UTR from the *Mus musculus* GRCm38.p5 assembly was retrieved from the Ensembl database release 86 and combined with the murine 3′UTRs from the rodent database in the UTRdb release 11 (44, 45). The software was run with strict 5’ seed pairing, energy threshold of −20 kcal/mol and default settings for gap open and gap extend penalties. Interacting hits were filtered by conservative cutoff values for pairing scores (>155) and matches (>80%). The resulting gene list was classified by the Panther classification system^2^ using pathway classification (46) and curated by the reactome pathway database^3^ (47). miRNA host target interactions to individual genes in cytokine pathways (PantherDB P00010, P00031, P00034, P00035, P00036, P00052, P00053, and P00054) of *Nb*-EV miRNAs, *Tm*-EV miRNAs (37), and shared homologs were linked and illustrated by the package “alluvial” v0.1-2 in R v3.3.2 (48).

### Exosome Uptake in Murine Small Intestinal (SI) Organoids (Mini-Guts)

Murine SI organoids were produced from intestinal crypts of a female C57 Bl6/J mouse according to previous reports (49) with some modifications. Briefly, murine SI crypts were dissociated with Gentle Cell Dissociation reagent (Stemcell Technology Inc., Vancouver, BC, Canada). Approximately 500 crypts were seeded in 50 µl of Matrigel (Corning Inc., New York, NY, USA) in a 24-well plate and cultured in Intesticult Organoid Growth Medium (Stemcell Technology Inc.).

Imaging was performed as described in Eichenberger et al. (37) with minor modifications. Briefly, to investigate internalization of EVs in the SI epithelium layer, 30–50 million PKH26 (Sigma-Aldrich) -labeled EVs in 3–5 µl were injected into the central lumen of individual organoids and cultured for 3 h at 37 and 4°C, respectively. Washed organoids were fixed and autofluorescence was quenched with 50 mM NH_4_Cl in PBS (for 30 min at RT) and 100 mM glycine in PBS (for 5 min). Cell nuclei were stained with Hoechst dye (Invitrogen, Carlsbad, CA, USA) and images were visualized on a laser scanning confocal microscope (Zeiss 780 NLO, Zeiss, Oberkochen, Germany). Confocal image deconvolution was performed in ImageJ using the plugins “Diffraction PSF 3D” for PSF calculation and “DeconvolutionLab” with the Tikhonov–Miller algorithm for 2D deconvolution (50).

### Experimental Model of Colitis

To assess the prophylactic impact of *N. brasiliensis* secreted products on experimental colitis in mice, we used the 2,4,6-trinitrobenzene sulfonic acid (TNBS; Sigma-Aldrich) method of acute inducible colitis. Weight-matched (18.86–21.31 g) 6-week-old male BALB/c mice were purchased from Animal Resources Centre, assessed for health and placed at random in groups of five animals per cage. All the experiments were repeated with the same number of mice in each group, resulting in independent duplicate experiments using the same groups. Mice were maintained at the JCU animal facility (Cairns campus) under normal conditions of regulated temperature (22°C) and lighting (12 h light/dark cycle) with free access to pelleted food and water in accordance with Australian animal rights and regulation standards.

One day prior to the induction of colitis, 20 µg of the test compounds in 200 µl PBS per mouse were administered intraperitoneal to 5 mice per group, whereas in a first approach 6 different groups were included in the study: (1) healthy naïve mice; (2) PBS (colitis control); (3) *Nb*-EVs; (4) *N. brasiliensis* ESP; (5) *N. brasiliensis* vesicle-depleted ESP (protein fraction); and (6) grapeosomes (vesicle and purification control). The experiment was repeated in an independent duplicate experiment (resulting in a total of 10 mice per group). *Tm* EVs and *Tm* vesicle-depleted ESP were evaluated in another, repeated experiment only.

TNBS colitis was induced as described earlier (33, 51). Animals were monitored daily for clinical signs including weight loss, piloerection, mobility, and fecal consistency/bleeding. An overall cumulative clinical score included weight loss (increase = 0; no weight loss = 1; loss = 2), piloerection (absent = 0; mild = 1; severe = 2), feces (normal = 0; mild diarrhea = 1; bloody, liquid, or unable to defecate after 5 min = 2), and mobility (normal = 0; lethargic = 1; motionless, sickly = 2). Clinical monitoring was performed by the same person at similar time points in a blinded manner (unaware of the groups). At day 3, mice were euthanized and the colon (from cecum to rectum) was removed and macroscopically assessed for colitis by scoring (absent = 0; mild = 1; moderate = 2; severe = 3) for the independent parameters of adhesions, ulceration, colonic thickening, and mucosal edema. Colon length was recorded, and 0.5–1 cm colon pieces were removed for *ex vivo* culturing for the measurement of tissue cytokine production and histological assessment of inflammatory infiltration. Tissue pieces for culturing were weighed to normalize cytokine data.

Colonic tissue was cultured in complete media (RPMI 1640, 10% heat-inactivated FCS, 1% HEPES, 100 U of penicillin/ml, 100 µg of streptomycin/ml, and 2 mM/l –glutamine; all reagents sourced from Invitrogen) for 24 h and supernatant was subsequently used to quantify levels of the cytokines IL-1β, IL-6, IL-10, IL17-a, IFN-γ, and TGFβ. Cytokine levels were measured by ELISA using Ready-Set-Go kits (Invitrogen) according to the manufacturer’s instructions, and a POLARstar Omega spectrophotometer (BMG Labtech, Thermo Fisher).

Tissue for histology was placed in formalin to fix tissue then transferred to 70% ethanol for storage and transport. Tissue was embedded in paraffin and sectioned longitudinally for histology. Slides were stained with hematoxylin and eosin (H&E). Tissue processing and staining was performed at the Cairns Hospital pathology laboratory. Inflammatory infiltrate was determined by the scoring method described in Hong et al. (52).

Results from the duplicate experiments were combined for statistical analysis. Statistical analyses were performed using GraphPad Prism (version 7.03). Comparisons were made between the sample treatment with TNBS groups and the PBS + TNBS group; *p* values of <0.05 were considered significant. When two groups were compared, a Mann–Whitney (unpaired, non-parametric) *U*-test was applied. All data are representative of at least two experiments (total *n* = 10 mice; with 5 mice/experimental group).

## Results

### *N. brasiliensis* Secretes EVs That Are Internalized by Host Cells

**Figure 1:**
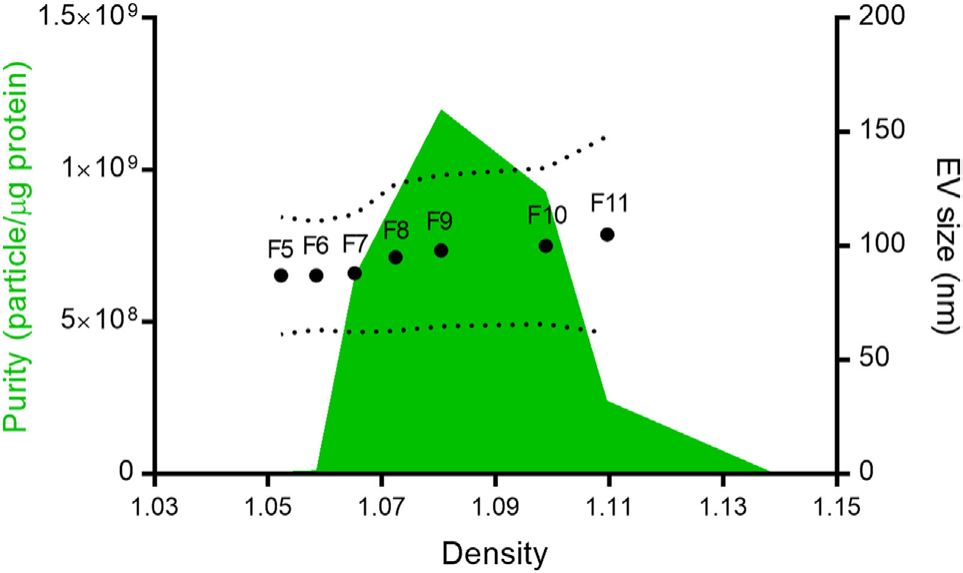
*Nippostrongylus brasiliensis* secreted extracellular vesicles (EVs). Mean particle size (dots), size range (dotted line), and purity (green area) of the different fractions isolated after Optiprep^®^ density gradient centrifugation. Despite protein being detected in all fractions, only vesicles from fractions 5–11 (F5–F11) could be quantified by tunable resistive pulse sensing. The purity of the different fractions was calculated according to Webber and Clayton (35).

**Figure 2:**
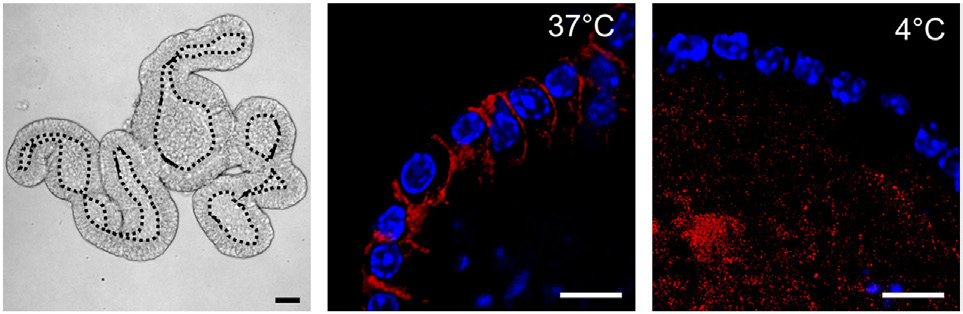
*Nippostrongylus brasiliensis* extracellular vesicles (EVs) are internalized by murine small intestinal (SI) organoid cells. Representative laser scanning confocal microscopy images (Zeiss 780 NLO) of PKH26-labeled EVs (red) at 37 and 4°C (metabolically inactive cells). EVs are internalized by cells within organoids at 37°C 3 h after particle-injection into the organoid central lumen (corresponding to the luminal side of the gut). Hoechst dye (blue) was used to label cell nuclei. Left panel demonstrates a bright field image (Zeiss AxioImager M1 ApoTome) of the tissue architecture of a murine SI organoid. Central lumen of the organoids is separated by the dotted line from the epithelial cell layer. Bar corresponds to 10 µm.

In 12 ODG fractions from concentrated and purified *N. brasiliensis* ESP, we purified vesicles in a size range of 60–160 nm (mean 95 ± 37.3 nm), which were most abundant in fractions 7–10 (density of 1.06–1.11 g/ml) as detected by qNano TRPS (Figure 1). *Nb*-EVs were verified by proteomic analysis, revealing several proteins which are frequently present in mammalian exosomes (“EV-markers”), including tetraspanin (NBR_0001199101), enolase (NBR_0001176401), 14-3-3 protein (NBR_0000671101), heat shock protein 70 (HSP70; NBR_0000494801), histones, and structural/cytoskeletal proteins (Table S1 in Supplementary Material). It has been demonstrated that EVs from *Tm* are actively internalized by murine intestinal cells within colonic organoids (37). We assessed whether murine host intestinal cells internalized *Nb*-EVs using murine small intestine (the site of residence of the adult worm) organoids, comprised of the complete census of progenitors and differentiated cells from the SI epithelial tissue growing in cell culture. We observed internalization of *Nb*-EVs by organoid cells cultured at 37°C but not at 4°C when cells were metabolically inactive and endocytosis was inhibited (Figure 2). Confocal microscopy images revealed that fluorescently labeled EVs were detected inside the cells with a cytoplasmic location within the donut-shaped organoid epithelial layer.

### *N. brasiliensis* but Not *Tm* EVs Protect Mice Against Chemically Induced Colitis

**Figure 3:**
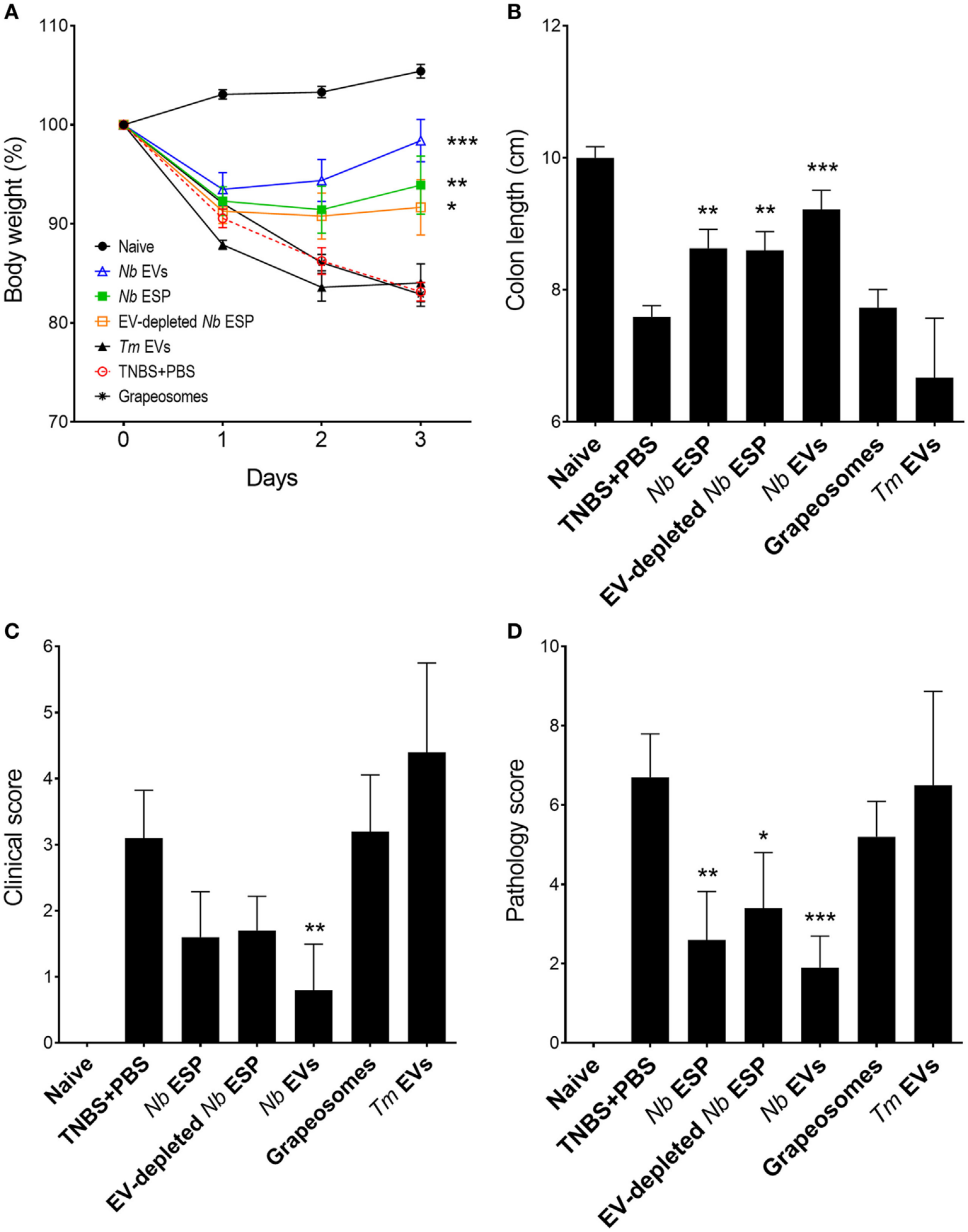
Protective effects of *Nippostrongylus brasiliensis* secreted fractions in experimental colitis. Mice received a single intraperitoneal injection of 20 µg protein in PBS 1 day prior to intrarectal administration of 2.5 mg of TNBS in 50% ethanol. **(A)** Body weight was recorded daily for the indicated groups. **(B)** Colon length measured after euthanasia at the end of the experiment (day 3). **(C)** Clinical examination and scoring of mice on day 3, including weight loss, piloerection, feces consistency, and mobility of mice from normal to severely affected (0–3). **(D)** After euthanasia, colons were visually scored by presence of adhesions, edema, mucosal wall thickening, and ulceration from absent to severe (high) on a scale of 0–3. Data show mean ± SEM of pooled data from two independent trials (*n* = 10 mice/group). Groups were compared to the PBS + TNBS (colitis) control group by Mann–Whitney *U*-test. *p < 0.05; **p < 0.01; ***p < 0.001. Data for *Trichuris muris* (*Tm*) extracellular vesicles (EVs) were analyzed in a separate independent experiment (see Figure S1 in Supplementary Material) and included only for representation.

The immunomodulatory properties of EVs from two distinct soil transmitted nematodes (rodent hookworm *N. brasiliensis* and whipworm *Tm*) were explored in experimental colitis. The chemically (TNBS)-induced mouse model of colitis is T-cell mediated and skewed toward a mixed Th1/Th2 immune response and induces transmural inflammation in the gut with clinical features similar to human ulcerative colitis (53). Interestingly, only secreted proteins and vesicles from *Nippostronglylus* (ESP, EVs, and vesicle-depleted ESP) showed efficacy in preventing colitis signs and symptoms, whereas purified fractions from *Tm* did not confer significant protection (Figure 3; Figure S1 in Supplementary Material).

Induction of intestinal inflammation resulted in a 15–20% weight loss in the PBS-treated colitis control group over the course of the study (Figure 3A). Mice from all groups initially lost weight, whereas *Nb*-EV-treated mice recovered most of their initial weight by the end of the experiment (on day 3). In comparison to the naïve healthy control mice, colon length was significantly decreased in the colitis group (*p* < 0.001), while *Nb*-EV-treated mice remained unaffected by the administration of TNBS.

**Figure 4:**
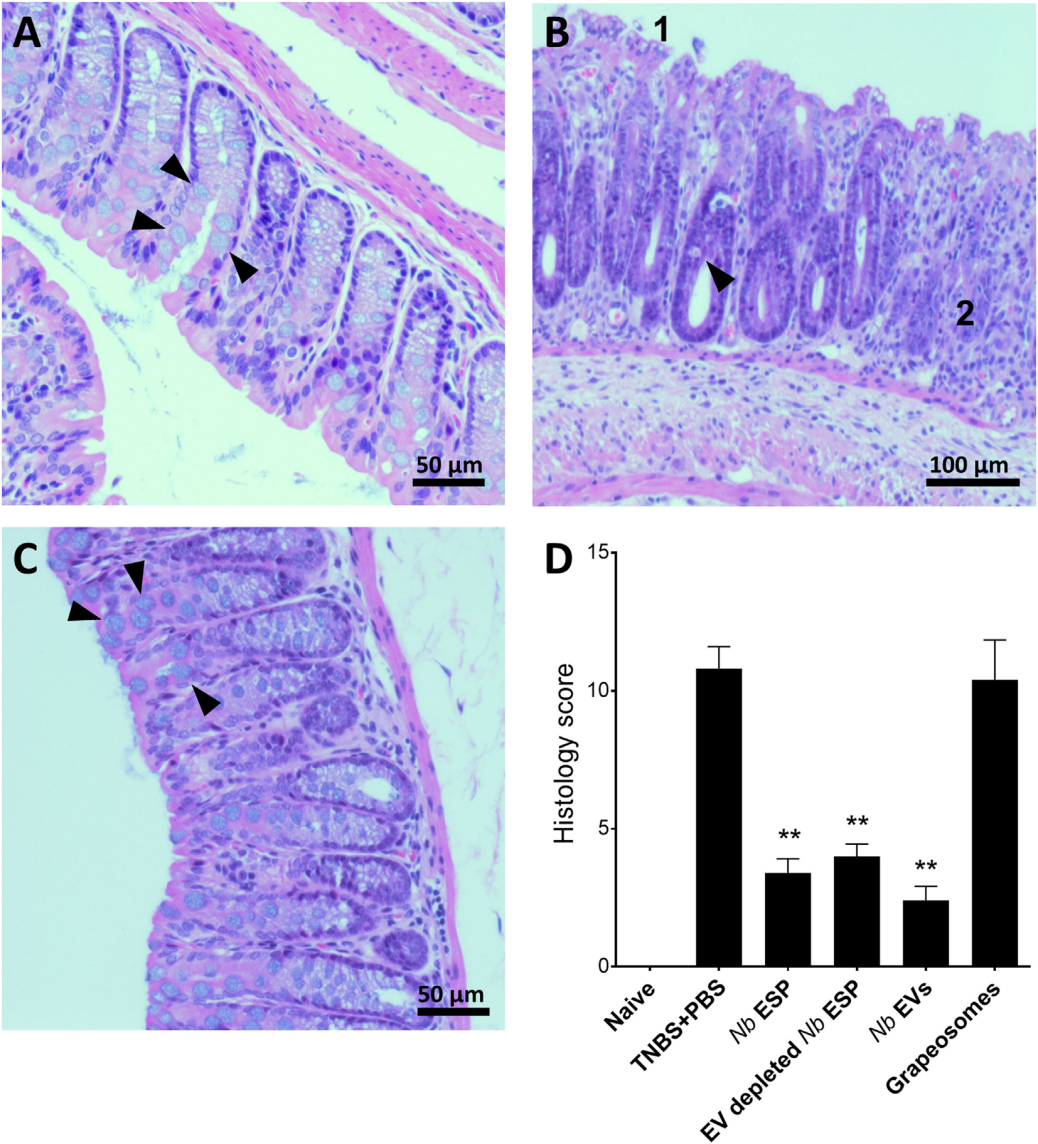
*Nippostrongylus brasiliensis* extracellular vesicles (EVs) protect from TNBS-induced histopathology. Representative photomicrographs of hematoxylin and eosin stained colonic tissue sections. A representative section from the distal colon (~1 cm) was fixed in 4% paraformaldehyde for histological observations. **(A)** Healthy naïve control mouse; **(B)** PBS-treated colitis control; **(C)** *N. brasiliensis* EV-treated mouse. Arrows point to normal goblet cells **(A,C)** and goblet cell destruction **(B)**. (1) Tissue erosion and (2) cellular infiltrates and overall tissue hyperplasia. **(D)** Histological scoring of histopathology. Statistical analyses were performed by pooling data from groups of mice from two independent but reproducible experiments (*n* = 10 mice/group). Error bars represent mean ± SEM. Groups were statistically compared to the PBS + TNBS (colitis) control group (**p < 0.01).

Macroscopic analysis of the colons revealed a significant reduction of tissue inflammation in animals treated with *Nippostrongylus* secreted fractions as seen by significant longer colons, fewer adhesions, absence of mucosal edema and colon wall thickening, and no ulceration (Figure S2 in Supplementary Material), reflected by significantly improved clinical and pathological scores (Figures 3C,D). Unlike *Nb*-EV-treated mice, histology (H&E staining) of distal colon sections from the PBS group showed mucosal erosion and epithelial hyperplasia, pronounced cellular infiltration in the lamina propria and intraepithelial compartments, evidence of edema and ulceration, and loss of healthy goblet cells (Figure 4B). Scoring of histological sections for overall pathology illustrated that *Nb*-EV-treated mice had significantly reduced histopathology (*p* = 0.004) (Figures 4C,D), displaying an overall mucosal architecture similar to that of naïve healthy control mice (Figures 4A,D).

Compared to other tested fractions from *Nippostrongylus, Nb*-EVs generally had the best scores in all of the tested parameters (weight loss, colon length, clinical-, macroscopic-, and histological score), although without statistical differences between the fractions. The purification method or the presence of vesicles itself did not have an impact on intestinal inflammation, as the grapeosomes-treated group showed severe inflammation post-TNBS administration, similar to that observed for the PBS + TNBS control group.

### *Nb*-EVs Promote Immune Regulation in Colonic Tissue Which Is Different From That Induced by Soluble ESP Proteins

**Figure 5:**
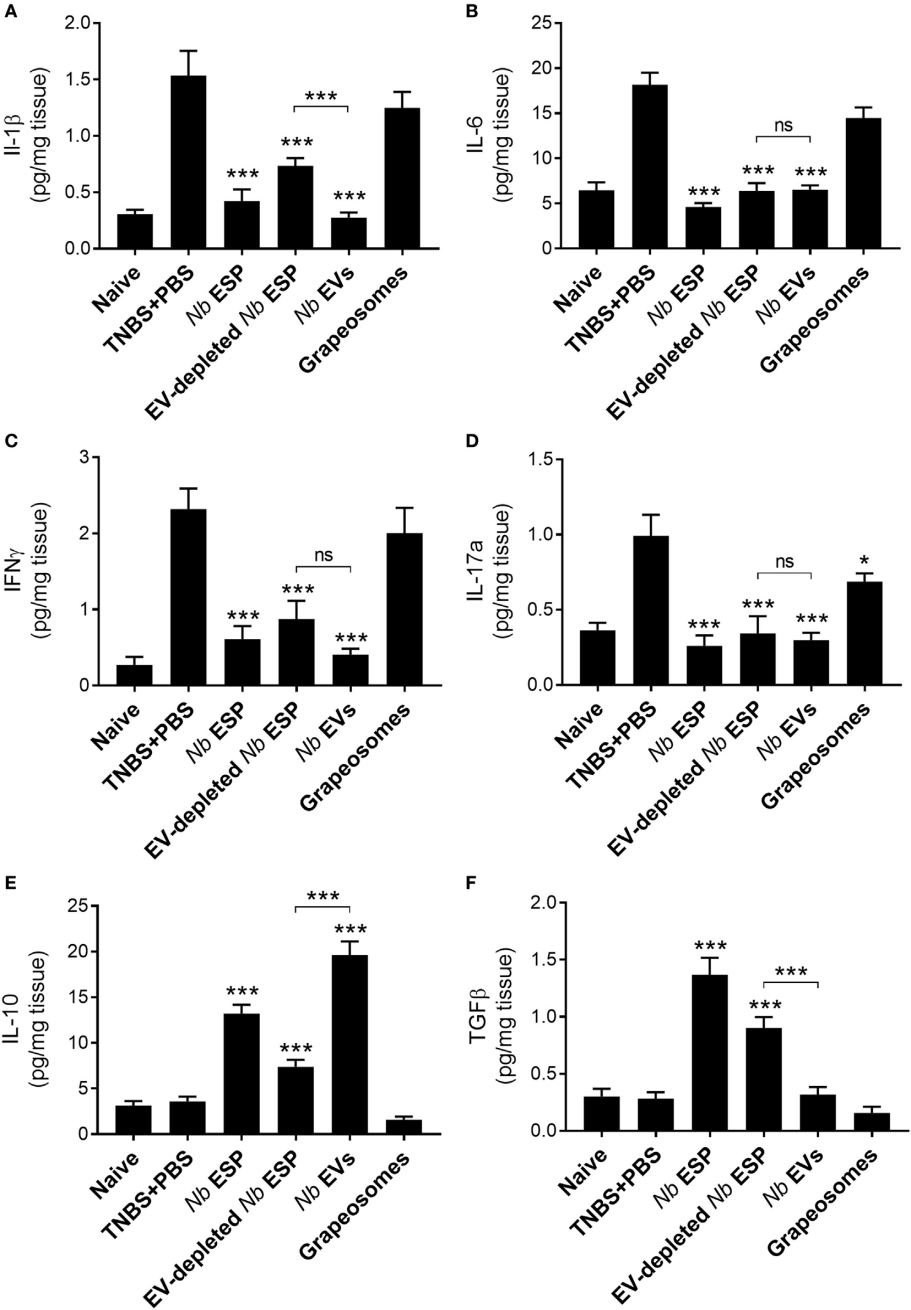
*Nippostrongylus brasiliensis* extracellular vesicles (EVs) suppress colonic inflammatory cytokine production and promote IL-10 secretion in mice. Cytokine profile of cultured colon tissue of healthy naïve mice, mice treated with *N. brasiliensis* secreted products, PBS colitis control and grape-vesicle (grapeosomes) control mice. **(A)** IL-1β; **(B)** IL-6; **(C)** IFNγ; **(D)** IL-17a; **(E)**; IL-10; and **(F)** TGFβ. All groups were compared to the PBS + TNBS (colitis) control group; the *N. brasiliensis* secreted protein group (EV-depleted) was also compared with the purified vesicles group (bracket). All comparisons were performed using a Mann–Whitney *U*-test. *p < 0.05; **p < 0.01; ***p < 0.001. Error bars represent mean ± SEM.

To address the impact of *Nippostrongylus* secreted molecules on the production of cytokines at the site of inflammation, colons of mice exposed to TNBS were cultured and cytokine secretion was analyzed by ELISA (Figure 5). Mice treated with any *Nippostrongylus* secretory product prior to administration of TNBS showed a significant reduction in the levels of the pro-inflammatory cytokines IL-1β, IL-6, IL-17a, and IFNγ, and the levels were—except for IL-1β in the secreted protein fraction—similar to those of naïve healthy control mice. By contrast, only the levels of the anti-inflammatory cytokine IL-10 was increased in the *Nb*-EV-treated group, which was significantly higher (*p* < 0.001) than that of mice treated with secreted proteins only. In comparison, the levels of TGFβ in the *Nb*-EV group were not significantly different from the healthy naïve mice or the PBS-treated group.

### *Nb*-EVs Contain Helminth-Specific Proteins and miRNA Cargo With Putative Immunomodulatory Properties

Purified *Nb*-EVs were digested with trypsin and analyzed by LC-MS/MS, resulting in a list of 81 proteins for *Nb*-EVs (Table S1 in Supplementary Material). Next to the proteomic “EV-markers” (*n* = 8) and structural proteins (*n* = 7) mentioned previously, the most abundant *Nb*-EV proteins (*n* = 27, 33.3%) were sperm-coating protein (SCP)-like extracellular proteins, also called SCP/Tpx-1/Ag5/PR-1/Sc7 domain containing proteins (SCP/TAPS), of which a high proportion (9 of 27) belonged to the helminth-specific *Ancylostoma*-secreted protein family (ASP; syn. activation-associated proteins). Furthermore, the dataset contains proteinases (*n* = 10), hypothetical proteins (*n* = 8), membrane-bound enzymes and transport proteins (*n* = 5), chaperones other than HSP70 (*n* = 2), and other metabolic enzymes (*n* = 13). In 53 (65.4%) of the 81 proteins, a signal peptide was absent (Table S1 in Supplementary Material), which is characteristic for EV proteins as a class of non-classically secreted particles.

Despite the differences between the two nematode EV populations in their immunological protection against colitis, their proteomic cargo share high sequence- and functional homology, including the abundantly represented SCP/TAPS proteins. Proteins unique for the *Nb*-EV dataset consist of seven uncharacterized hypothetical proteins, three apyrase isoforms—which are catalysts for the hydrolysis of ATP to yield AMP and inorganic phosphate—and a saposin protein (Table S2 in Supplementary Material).

**Figure 6:**
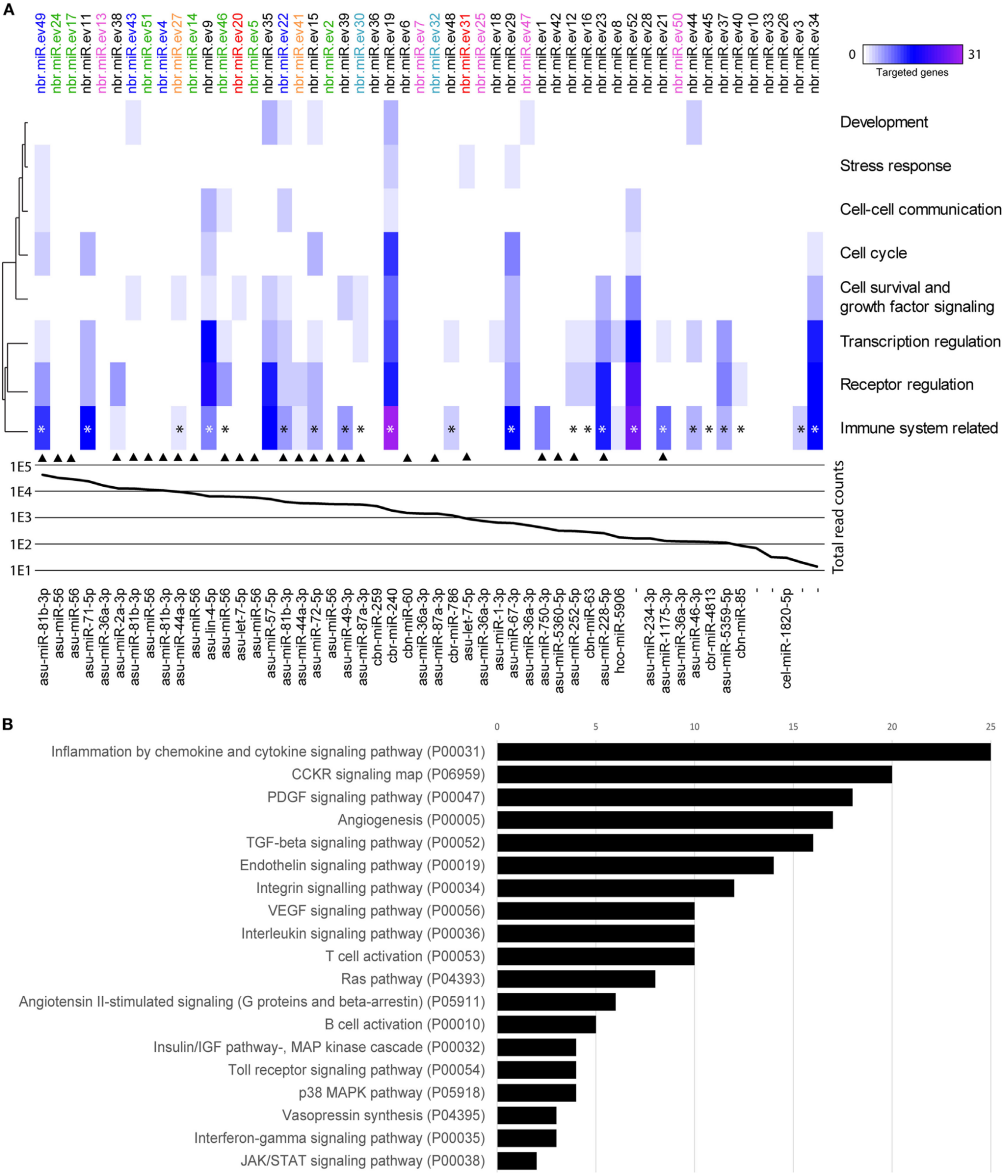
miRNAs of *Nippostrongylus brasiliensis* extracellular vesicles (EVs) are predicted to interact with different murine host gene networks. **(A)** Functional map of *N. brasiliensis* EV miRNAs and their target murine host genes categorized by PantherDB signaling pathway analysis (heat map corresponds to individual targeted genes in the murine host). Top axis shows the 52 identified miRNAs (termed as nbr-miR-ev#). IsomiRs are indicated in color. Graph represents the abundance (mean read counts from three biological replicates). Bottom axis shows their closest homologs (*de novo* transcripts are left empty). Homologs to miRNAs found in *Trichuris muris* EVs are marked by an arrow [according to Eichenberger et al. (37)]. * indicates miRNAs targeting genes involved in cytokine networks. **(B)** Total number of targeted gene networks identified by PantherDB categories classified as “immune system related.” Data are available in Table S3 in Supplementary Material.

By sequencing and screening biological triplicates for miRNA cargo in *Nb*-EVs using the Illumina NextSeq platform and downstream analyses, we identified 52 miRNAs commonly present in all datasets, 47 of which have close homologs to 31 other nematode miRNAs (Figure 6).

Potential interactions of *N. brasiliensis* miRNAs with murine host genes were explored by computational target prediction. The 52 nematode EV-miRNAs were predicted to interact with 2,093 unique 3′UTR binding sites of the mouse genome assembly (Table S3 in Supplementary Material). Associated annotated coding genes were grouped according to signaling, metabolic, and disease pathways (Figure S3 in Supplementary Material). Interestingly, immune system-related gene networks were predicted to be targeted by 30 of the 52 detected miRNAs, of which 23 directly affect cytokine signaling networks—including the most abundant nbr-miR-ev49 (Figure 6; Table S4 in Supplementary Material).

Given that *Nb*-EVs but not *Tm-*EVs protected against inducible colitis in mice, we compared the vesicular miRNA cargo of these two nematodes. When we compared the miRNA component of *Nb*- and *Tm-*EVs, we found only 26 Nbr_miRs to be homologous to *Tm*-EV miRNAs, some of which are isomiRs (Figure 6). Of these, 13 shared miRNAs were predicted to target gene networks involved in the immune system (10 of which targeted cytokine gene networks). We further analyzed the miRNA host gene target prediction for specific interactions with genes involved in pro- and anti-inflammatory cytokine responses based on the miRanda algorithm (Figure S4 in Supplementary Material). This global cursory analysis of host gene interactions by nematode EVs points to a strong regulation of cytokine gene networks through parasite miRNAs. The analysis illustrates that EV-miRNAs from both nematodes interact with pro- and anti-inflammatory host genes. Overall, however, there are more cytokine genes targeted by EV miRNAs that are unique to *N. brasiliensis* (*n* = 29) than by EV miRNAs that are unique to *Tm* (*n* = 17). Prediction analyses unfortunately are not able to demonstrate the fate of the targeted gene (i.e., upregulated vs. downregulated expression).

## Discussion

Immune evasion is a common strategy of parasitic helminths to survive and reproduce within a hostile environment, while neutralizing immune pathways that would otherwise expel them and resetting the thresholds of immune reactivity (15). Hookworms have evolved to establish chronic infections while inducing minimal pathology to the host when present in small numbers (3). They achieve this state of mutual tolerance by promoting regulatory immune circuits *via* expansion of various regulatory and tolerogenic immune cell subsets (3, 15). Hookworms drive a “modified Th2” immune response, including typical Th2 cytokines (IL-4, IL-5, IL-9, and IL-13) but also the regulatory cytokines IL-10 and TGF-β. In hookworm-infected humans at least, the skewing of the immune response enables the parasite to survive for long periods, despite the presence of a robust, albeit non-sterilizing immune response (54–56). Hookworms and humans have instead coevolved to reach an immunological *status quo*, where Th2 responses likely keep worm burdens in check (so as not to overwhelm and ultimately kill the host), but regulatory responses ensure that at least some worms survive and reproduce over many years.

The immunoregulatory prowess of hookworms has been highlighted in clinical trials for IBD (57) and celiac disease (20). Using animal models of inflammatory diseases, we and others have shown using *N. brasiliensis* (58) and *Ancylostoma* sp. (21–23, 25) that injection of ESP alone mimics the immune phenotype of the worm infection and is sufficient to suppress inflammation in numerous models of autoimmunity and allergy. Until now, identification of bioactive hookworm ESP molecules has placed emphasis on the protein moieties (25, 33, 59), and other molecular entities have been ignored.

As we show herein, a major component of ESP from hookworms and other helminths is EVs. These parasite EVs have been shown to deposit their payloads consisting of proteins, nucleic acids, lipids, and metabolites into host cells where at least some of them exert their immunomodulatory properties (27, 28). Here, we demonstrate that *N. brasiliensis*, which is frequently used as a model for human hookworm infection, secretes exosome-like EVs that possess immunoregulatory molecules.

Extracellular vesicles from the trematode *Opisthorchis viverrini* and the nematodes *Brugia malayi, Heligmosomoides polygyrus*, and *Tm* are internalized by host cells (32, 37, 60, 61). Similarly, *Nb*-EVs interact with murine cells, as demonstrated by the uptake of stained EVs *in vitro* in murine SI organoids. Similar to *Tm*, the cellular interaction seems to be non-specific, and all cell types found within the organoids (mainly absorptive enterocytes, goblet cells, enteroendocrine cells, Paneth cells, and Lgr5+ stem cells) contained fluorescently labeled EVs. One drawback of this groundbreaking organoid culture system (62) is the lack of immune cells. Hence, further studies are needed to explore the specific impact of parasite EVs on host immune cells, particularly T cells (e.g., intraepithelial lymphocytes) and antigen-presenting cells.

To evaluate the immunomodulatory properties of nematode EVs, we induced T cell-dependent acute colitis in mice. In this model, TNBS haptenizes the colonic microbiota, which then translocates across the ethanol-disrupted gut epithelium and elicits a mixed Th1/Th2 immune response, and induces transmural inflammation in the gut with clinical, morphological, and histopathological features similar to those of human IBD (63). Our results indicate that EVs from *Nippostrongylus* (*Nb*-EVs) protected against intestinal inflammation, whereas EVs from *Tm* did not. This finding is somewhat surprising, given that *Trichuris* spp. have coevolved with their hosts to establish chronic infections. A major difference in the biology of these two GI helminths is the life cycle—hookworm infect the host by skin penetration followed by a refined systemic migration through the vasculature of the lungs en route to the small bowel whereupon they bury their anterior ends in the sub-mucosa and feed on extravasated blood; whipworms, however, have a direct oral infection route and feed on (and burrow into) the epithelial layer.

Prophylactic treatment of mice prior to administration of TNBS with *Nb*-EVs, “complete” ESP, or the EV-depleted soluble protein fraction, resulted in suppression of pro-inflammatory cytokines IFN-γ, IL-6, IL-17a, and IL-1β. IL-10 has a protective role against colitic inflammation (64). Furthermore, genetic-linkage analysis of patients with colitis revealed distinct mutations in the IL-10 gene, demonstrating a central role for this cytokine in the negative feedback necessary to maintain mucosal homeostasis (65, 66). As seen previously with “complete” *Ancylostoma caninum* (dog hookworm) ESP treatment in TNBS colitis (21), *Nb*-EVs promoted the production of IL-10, suggesting a potential mechanism of systemic regulation of inflammation. By contrast, TGF-β was found to be elevated only in mice which were treated with secreted proteins (ESP or vesicle-depleted fraction) and not EVs. Nevertheless, these groups displaying elevated TGF-β levels, although significantly protected against some parameters of colitis, generally displayed lower levels of protection compared with EV-treated mice. TGF-β is responsible for suppression of gut inflammation and enhancing barrier function, and it promotes the induction of functional Tregs from naive CD4+ T-cell precursors (67, 68). Hence, nematode immune evasion strategies rely most probably on a finely tuned cocktail of soluble and vesicular molecules to regulate host immunity.

The EV proteomes of both *N. brasiliensis* and *Tm* are replete with SCP/TAPS proteins. This family of proteins is abundantly expressed by parasitic nematodes and trematodes (69). Their roles are still mostly unknown, but in hookworms they have been suggested to play roles in larval skin penetration (70), in the transition from the free-living to parasitic stages (71), and modulation of the immune response (72, 73). Despite the significantly greater ability of hookworm EVs compared with whipworms EVs to suppress TNBS-induced inflammation, SCP/TAPS proteins were over-represented in both EV populations.

An emerging mechanism of parasite-driven immune modulation is *via* the transfer of genetic information between host and parasite. To this end, we identified 52 *N. brasiliensis* miRNAs, including five novel miRNAs without homology to other nematode miRNAs deposited in the reference database. miRNAs are considered as regulators of the immune response by targeting host immune cell mRNAs for degradation or translational repression (74). *N. brasiliensis* miRNAs that putatively regulate expression of mouse genes involved in specific gene networks and cellular pathways were identified. Our *in silico* prediction analysis of murine host gene interactions of miRNAs points toward a strong involvement of parasite miRNAs in regulation/modulation of the host immune system. Although there were few homologies with recently published *Tm* EV miRNA datasets (37, 75), the *N. brasiliensis* miRNAs seem to target immunological networks more specifically *via* a greater abundance and redundancy of several isomiRs. The prediction resulted in a potential 2,093 unique interactions with mouse transcripts. Although it is known that target-predictions bear a high false-positive rate, it provides insights into the most highly rated interaction networks. Correspondingly, the most affected pathway was “cytokine and chemokine signaling” (P00031 in PatherDB). The located genes encoded mostly chemokine receptors and downstream signaling molecules (data not shown but mined from Table S3 in Supplementary Material). Interestingly, pathway analysis indicated that *Nb*-EV miRNAs mapped to interleukin-networks, notably IL-6 receptor and IL-6 signal transducers, IL-17 receptor genes, and IL-21. We also identified single hits with interactions to the Th2 cytokines IL-13 and IL-33, many of which showed altered expression in EV-treated mice after TNBS administration. Furthermore, targeting of the IFNγ- (P00035) and TFGβ- (P00052) signaling pathways was noted. Overall, our analysis of putatively targeted host genes illustrates that EV-miRNAs from nematodes interact with pro- and anti-inflammatory pathways. Interestingly, *Tm*-EV miRNAs seem to primarily target pathways that function downstream of cytokine receptor engagement, such as signal transduction (e.g., IRAK2/4) and transcription factors (e.g., STAT1, NFATs, and SMADs), while *Nb*-EV miRNAs directly target cytokine and cytokine receptor transcripts. However, *in vivo* experiments with miRNAs (separated from other EV components such as proteins) are needed to confirm the *in silico* predictions.

Although not explored herein, regulation of angiogenesis and wound-repair mechanisms (e.g., vascular endothelial growth factor; P00056) were frequently targeted by *Nb*-EV miRNAs. Given that miRNAs, in addition to their well-studied repressive function, can act with certain context-dependent factors to stabilize and increase translation of targets by both transcriptional and posttranscriptional mechanisms (76), a role for worm EVs in healing and vascularizing the wounds it causes when feeding is plausible (77).

In summary, *Nb*-EVs induced protection against intestinal inflammation while EVs from an unrelated source (grapes) did not. By contrast, EVs from the whipworm *Tm* did not induce protection against acute colitis. Administration of *Nb*-EVs to mice induced a unique cytokine profile compared to that induced by soluble ESP proteins; *Nb*-EVs promoted significantly greater levels of IL-10 secretion in the colon compared to soluble EV-depleted ES products, and this finding might be due to miRNAs contained within the EVs. Our findings provide insight into the immunobiology of hookworm EVs, and show for the first time that helminth EVs suppress colitis and likely harbor therapeutic molecules for the treatment of inflammatory bowel and other autoimmune diseases.

## Ethics Statement

The study was approved by the James Cook University (JCU) Animal Ethics Committee (A2180, A2213, and A2300). Animals were maintained at the JCU animal house (Cairns campus) under normal conditions of regulated temperature and lighting (12 h light/dark cycle) with free access to pelleted food and water. Mice and rats were kept in cages in compliance with the Australian Code of Practice for the Care and Use of Animals for Scientific Purposes.

## Author Contributions

RE, JS, and AL conceived and designed the study. RE performed most of the experiments. SR, LJ, RP, GB, and JS assisted in the *in vivo* experiments and analyses. MO and CE facilitated imaging. JZ performed sample histology. PG and LD propagated animal model and *in vivo* studies. MF provided bioinformatics assistance and support. RE and AL wrote the manuscript. All authors proofed the manuscript.

## Conflict of Interest Statement

The authors declare that this research was conducted in the absence of any commercial or financial relationships that could be construed as a potential conflict of interest.

## Footnotes

^1^http://www.thegpm.org/crap/ (Accessed: April 5, 2017).

^2^http://pantherdb.org/ (Accessed: December 7, 2017).

^3^www.reactome.org (Accessed: December 7, 2017).

## Supplementary Material

Figure S1 Effects of *Trichuris muris* secreted fractions in experimental colitis. Mice received a single intraperitoneal injection of 20 µg protein in PBS 1 day prior to intrarectal administration of 2.5 mg of TNBS in 50% ethanol. Data display daily recorded body weight, final clinical examination, colon length, and colon pathology after euthanasia.

Figure S2 Representative images of mouse colons from all the groups examined in the experimental colitis model.

Figure S3 Prediction of *Nippostrongylus brasiliensis* extracellular vesicle (EV) miRNA target interactions to murine host genes. Functional map of *N. brasiliensis* EV miRNAs and their target murine host genes categorized by PantherDB signaling, metabolic, disease, and other pathways. Heat map corresponds to individual targeted genes in the murine host.

Figure S4 Alluvial diagram depicting interactions between nematode EV miRNAs and mouse host cytokine gene targets. *Nippostrongylus brasiliensis* (*Nb*)-EV miRNAs (green), *Trichuris muris* (*Tm*)-EV miRNAs (pink), and shared homologs (orange) are presented. Links are colored according to canonical pro- (red) and anti-inflammatory (blue) responses.

Table S1 Proteomic analysis of extracellular vesicles (EVs) secreted by *Nippostrongylus brasiliensis*. Details of the identification of the proteins present in the EVs secreted by *N. brasiliensis* using X!Tandem, Tide, MS-GF+ and OMSSA. All proteins are shown, including contaminants.

Table S2 Structural (all-vs-all blast) and functional (Blast2GO) comparison of *Nippostrongylus brasiliensis*- and *Trichuris muris*-EV proteomes.

Table S3 Data description on predicted *Nippostrongylus brasiliensis* miRNA–host target interactions. Table showing the 52 miRNAs identified in the *N. brasiliensis* extracellular vesicles and their 3′UTR predicted binding sites in the mouse genome.

Table S4 Pathway analysis and parsing of *Nippostrongylus brasiliensis* EV miRNAs and their target murine host genes categorized by PantherDB pathways.
