# Supplementary figures and images for: Hookworm Secreted Extracellular Vesicles Interact With Host Cells and Prevent Inducible Colitis in Mice
Source: Front Immunol. 2018 Apr 30;9:850. doi: 10.3389/fimmu.2018.00850 (PMC5936971; doi:10.3389/fimmu.2018.00850)

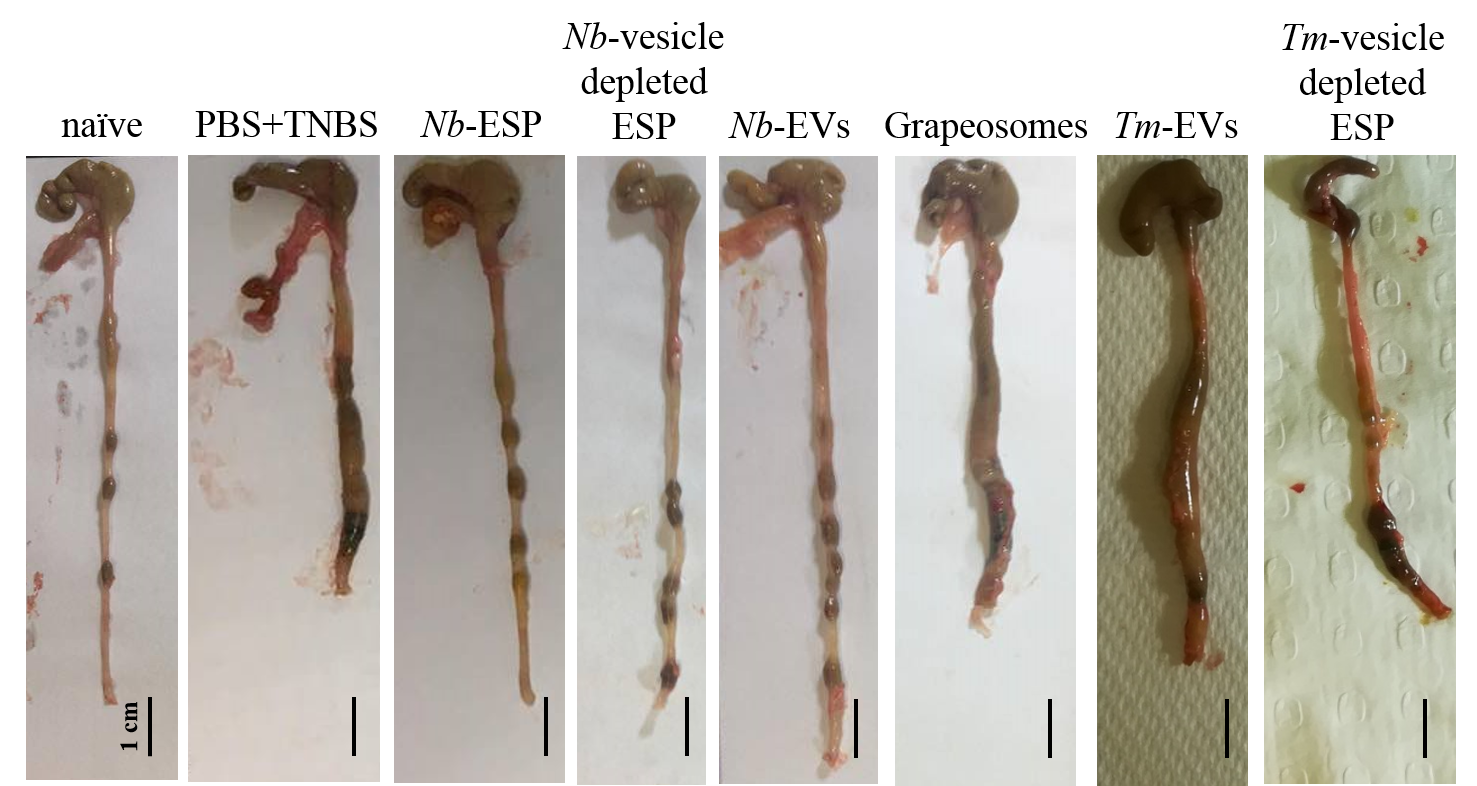

Supplement: Figure S2 — Representative images of mouse colons from all the groups examined in the experimental colitis model. [file image_2.tif]

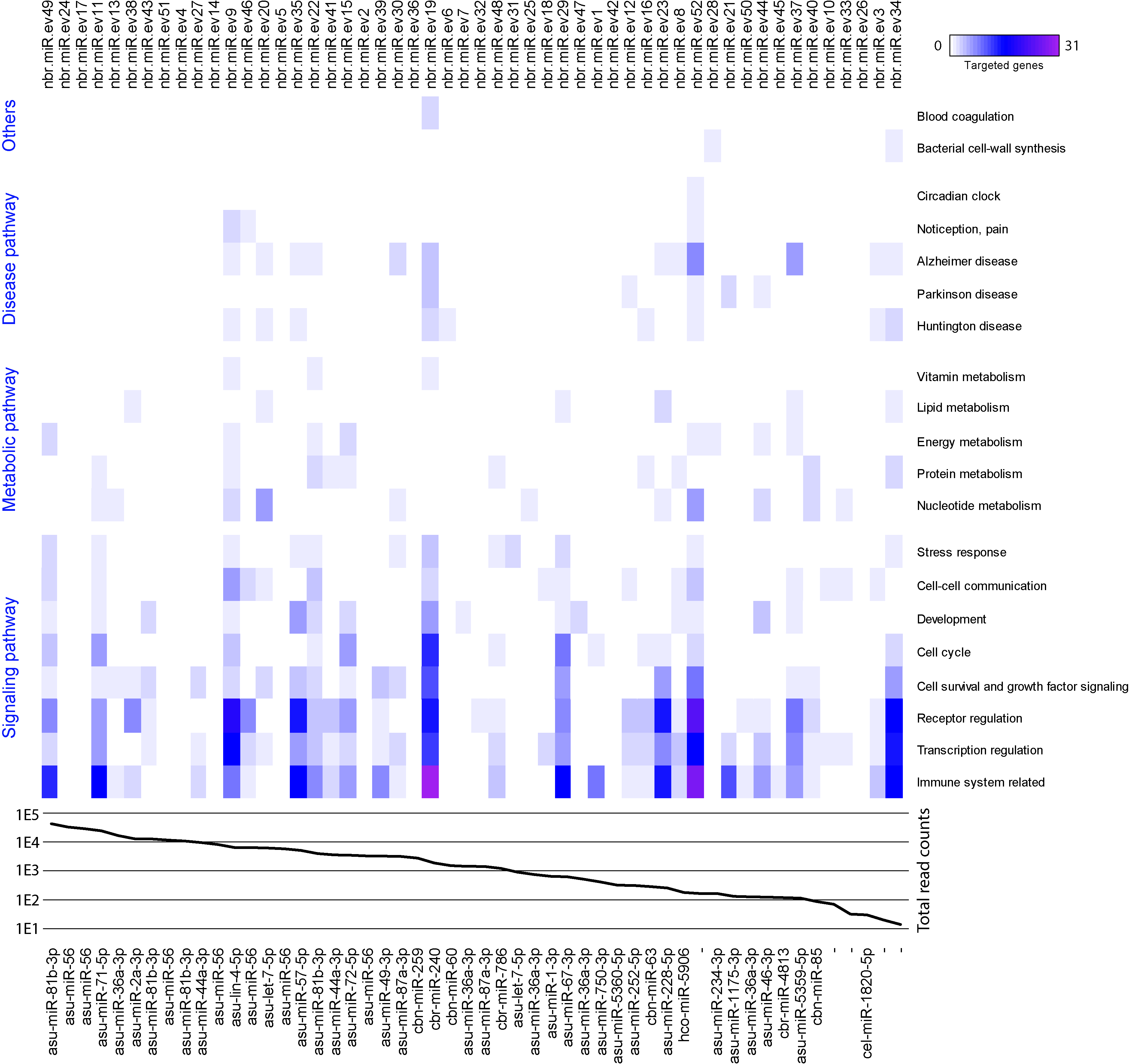

Supplement: Figure S3 — Prediction of Nippostrongylus brasiliensis extracellular vesicle (EV) miRNA target interactions to murine host genes. Functional map of N. brasiliensis EV miRNAs and their target murine host genes categorized by PantherDB signaling, metabolic, disease, and other pathways. Heat map corresponds to individual targeted genes in the murine host. [file image_3.tif]

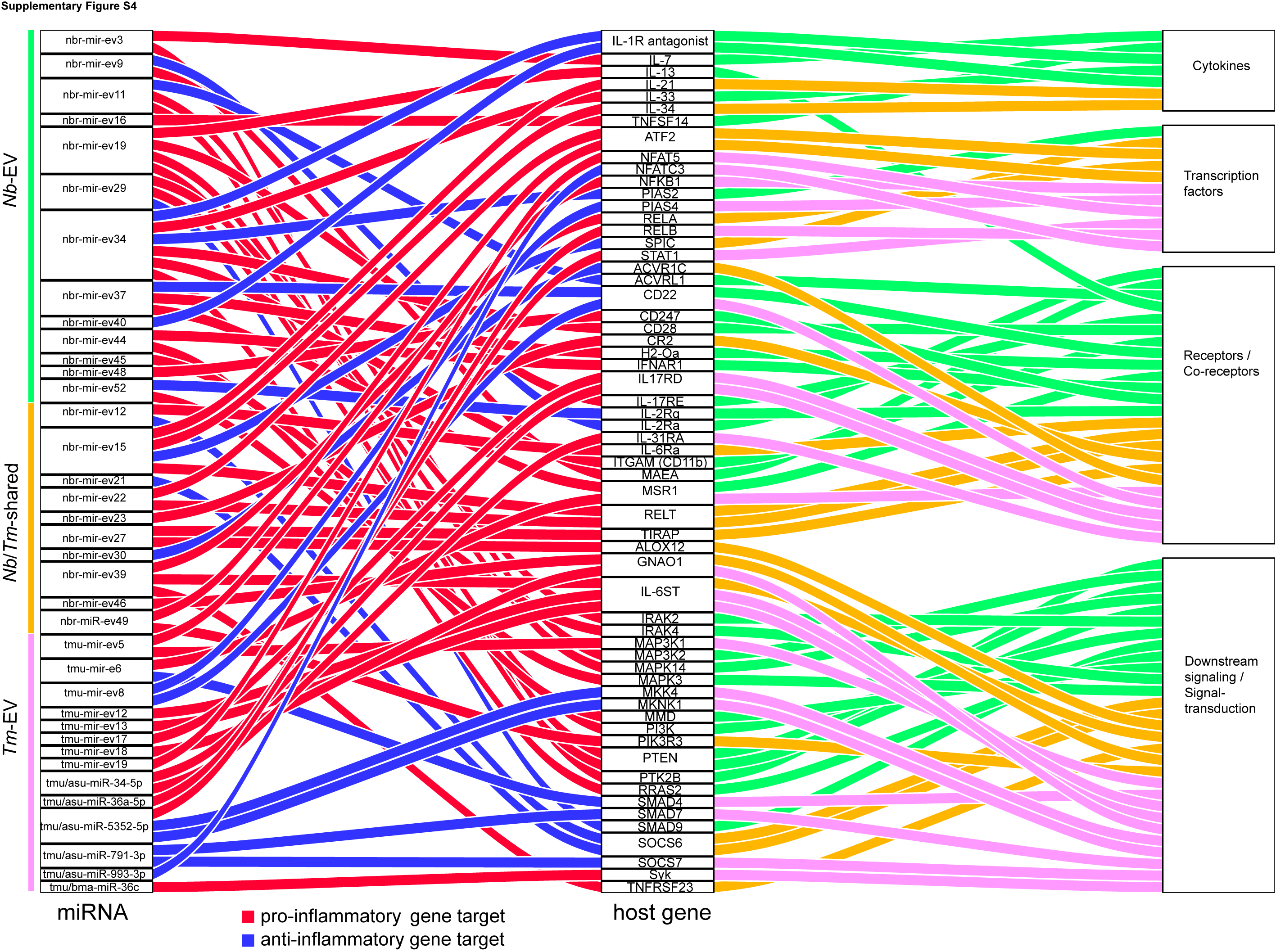

Supplement: Figure S4 — Alluvial diagram depicting interactions between nematode EV miRNAs and mouse host cytokine gene targets. Nippostrongylus brasiliensis (Nb)-EV miRNAs (green), Trichuris muris (Tm)-EV miRNAs (pink), and shared homologs (orange) are presented. Links are colored according to canonical pro- (red) and anti-inflammatory (blue) responses. [file image_4.tif]
